# Serum sodium variability and acute kidney injury: a retrospective observational cohort study on a hospitalized population

**DOI:** 10.1007/s11739-020-02462-5

**Published:** 2020-08-09

**Authors:** Gianmarco Lombardi, Pietro Manuel Ferraro, Alessandro Naticchia, Giovanni Gambaro

**Affiliations:** 1 U.O.C. Nefrologia, Azienda Ospedaliera Universitaria Integrata di Verona, Verona, Italy; 2 U.O.C. Nefrologia, Fondazione Policlinico Universitario A. Gemelli IRCCS, Via G. Moscati 31, 00168 Rome, Italy; 3 Università Cattolica del Sacro Cuore, Rome, Italy

**Keywords:** Acute kidney injury, Electrolyte disorders, Hyponatremia, Hypernatremia, Epidemiological study

## Abstract

Aim of our study was to analyze the association between serum sodium (Na) variability and acute kidney injury (AKI) development. We performed a retrospective observational cohort study on the inpatient population admitted to Fondazione Policlinico Universitario A. Gemelli IRCCS between January 1, 2010 and December 31, 2014 with inclusion of adult patients with ≥ 2 Na and ≥ 2 serum creatinine measurements. We included only patients with ≥ 2 Na measurements before AKI development. The outcome of interest was AKI. The exposures of interest were hyponatremia, hypernatremia and Na fluctuations before AKI development. Na variability was evaluated using the coefficient of variation (CV). Multivariable Cox proportional hazards and logistic regression models were fitted to obtain hazard ratios (HRs), odds ratios (ORs) and 95% confidence intervals (CIs) for the association between the exposures of interest and AKI. Overall, 56,961 patients met our inclusion criteria. During 1541 person-years of follow-up AKI occurred in 1450 patients. In multivariable hazard models, patients with pre-existent dysnatremia and those who developed dysnatremia had a higher risk of AKI compared with patients with normonatremia. Logistic models suggested a higher risk for AKI in the 3rd (OR 1.41, 95% CI 1.18, 1.70, *p* < 0.001) and 4th (OR 1.53, 95% CI 1.24, 1.91, *p* < 0.001) highest quartiles of Na CV with a significant linear trend across quartiles (*p* trend < 0.001). This association was also independent from Na highest and lowest peak value. Dysnatremia is a common condition and is positive associated with AKI development. Furthermore, high Na variability might be considered an independent early indicator for kidney injury development.

**Electronic supplementary material:**

The online version of this article (10.1007/s11739-020-02462-5) contains supplementary material, which is available to authorized users.

## Introduction

Electrolyte disturbances are common disorders in the hospitalized population [1]. Serum sodium (Na) imbalance is frequently observed in the hospital setting [2]. Dysnatremia conditions (including hyponatremia [Na < 135 mEq/L] and hypernatremia [Na > 145 mEq/L] are reported in approximately 30–40% of all hospital admissions [3].

Medical and scientific interest on these conditions is justified by the significant burden of Na disorders on the patient’s prognosis [4]. Both hyponatremia and hypernatremia have been widely associated with increased morbidity and mortality. Furthermore, as suggested by recently published studies, even small fluctuations in serum Na levels have been associated with a significant increase of in-hospital mortality [5–8].

As the main organ involved in water metabolism and homeostasis, the kidney is generally the main culprit for such disorders. Defective urine dilution with disproportionally high water intake causes hyponatremia. On the other way around, disorders involving urine concentration with inadequately low water intake cause hypernatremia [9, 10]. Therefore, it is not surprising that kidney diseases, especially acute kidney injury (AKI), characterized by an abrupt reduction in renal function, are commonly associated with these pathological conditions [11–15].

On the other hand, as sparsely reported in medical literature, an inverse relationship between dysnatremia and AKI emerges, where Na imbalance precedes and predicts kidney damage. A plausible cross-talk on a biological and pathogenetic ground might justify such relationship [16–18], but it still remains poorly investigated [14, 19].

The aim of our study is to analyze the association between dysnatremia, in the whole range of its manifestations (hyponatremia, hypernatremia and mild Na fluctuations in the normonatremic range), and AKI development using a large retrospective cohort of hospitalized patients.

## Methods

### Setting and study population

We performed a retrospective observational study on the hospitalized population admitted to Fondazione Policlinico Universitario A. Gemelli IRCCS, a tertiary level hospital serving more than 1 million people in Rome, between January 1, 2010 and December 31, 2014. We included only adult patients (aged 18 years or older) with at least two serum Na (with consensual serum glucose) and at least two serum creatinine measurements during hospital stay. For analysis and data calculation we included only patients with at least two Na measurements before AKI development. Patients with end-stage kidney disease (ESKD) were excluded. Study patients were included at the time of their first hospital admission. If a patient was hospitalized multiple times during the study period, we considered only the first one.

### Data collection

All data were extracted from the hospital electronic database. We exported the following demographic, clinical and laboratory data: age, sex, serum Na, glucose, creatinine, primary and secondary ICD-9-CM (International Classification of Disease, 9th Revision, Clinical Modification) diagnosis codes at hospital discharge.

### Definitions

Acute kidney injury (AKI) was defined according to creatinine kinetics criteria [20].

In-hospital AKI was defined as AKI developed after ≥ 48 h from hospital admission.

Patients were grouped according to all Na values recorded during hospital stay and preceding AKI development in the following dysnatremic groups: hyponatremia (Na value < 135 mmol/L), hypernatremia (Na value > 145 mmol/L), normonatremia (lowest/highest Na values ≥ 135 mmol/L and ≤ 145 mmol/L). In patients with mixed dysnatremia, only the first Na disorder (what happened first), hypo or hypernatremia, was considered.

All Na levels were corrected for the dilutional effect associated with hyperglycemia using a validated method [21].

Na variability (or fluctuations) was evaluated using the coefficient of variation (CV), defined as the ratio between the standard deviation and the mean of all Na values preceding AKI development.

Comorbid conditions (cardiovascular diseases, malignancies, gastrointestinal diseases, genitourinary disorders, endocrine/metabolic disorders, infectious and respiratory diseases) were identified using ICD-9-CM codes. The Charlson/Deyo comorbidity index score [22] was calculated for each hospital admission using primary and secondary diagnosis ICD-9-CM codes at hospital discharge.

ESKD was identified using administrative data (ICD-9 CM codes using chronic ESKD criteria: procedure codes for arteriovenous fistula creation or revision (39.27, 39.42, 39.43, and 39.93); history of ESKD requiring either kidney transplant (identified through discharge diagnosis ICD-9 V42.0) or maintenance dialysis (ICD-9 V45.1, V45.11, V45.12, V56, V56.0, V56.8). Dialysis criteria were: any of the following procedure codes: 39.95 (hemodialysis), V45.1 (renal dialysis status), V56.0 (extracorporeal dialysis), or V56.1 (fitting and adjustment of extracorporeal dialysis catheter); the initiation of dialysis in a patient with no known history of prior dialysis (ICD-9p 39.95, 54.98)) or laboratory data (baseline estimated glomerular filtration rate [eGFR] < 15 mL/min/1.73 m^2^). The baseline GFR was estimated for each hospital admission with the CKD-EPI formula [23] using the first creatinine value read at hospital admission.

### Outcomes and covariates

The outcome of interest was in-hospital AKI development.

The exposures of interest were the dysnatremia groups and Na fluctuations (expressed as quartiles of CV and analyzed as categorical and numeric variable).

The covariates that were used for risk adjustment in multivariable regression analyses were: age, sex, Charlson/Deyo score, comorbidities, Na value at hospital admission, eGFR at hospital admission. Survival time was defined as the time from in-hospital admission to primary outcome, loss to follow-up or censoring, whichever occurred first.

### Statistical analysis

Categorical variables were expressed as numbers and percentages. Continuous variables were expressed as means with standard deviations (SDs) (normal distribution) or medians with interquartile ranges (IQRs) (skewed distribution). Normality of distributions was evaluated by visual inspection of histogram and Q-Q plot.

To explore the association between dysnatremia and AKI, we used a cause-specific regression hazard model. To confirm our results, as sensitivity analysis, we performed subdistribution hazard models considering in-hospital death as competitive risk. Cumulative acute kidney outcome was obtained using the cumulative incidence function for competitive risk. Time at risk started when Na disorder (hypo- or hypernatremia) was first observed, in normonatremic patients at first Na measurement. All alive patients were censored at the time of hospital discharge. Unadjusted and multivariable adjusted hazard ratios (HRs) with 95% confidence interval (95% CI) were reported for all survival analyses.

A logistic regression model, unadjusted and adjusted for all covariates, was fitted to obtain the odds ratios (ORs) and 95% CI of the association between the quartiles of Na CV and in-hospital AKI. Three models were built: Model 1 represents unadjusted ORs; Model 2 was adjusted for age, sex, comorbidities, Na value at hospital admission, baseline eGFR; Model 3 was adjusted for Na lowest and highest peak value in addition to factors included in Model 2. The first CV quartile was used as reference. A *p* value for trend was calculated by treating quartiles as continuous variables in each model.

To evaluate the modification effects of subgroups on the relationship between dysnatremia and AKI development, a preplanned subgroup analysis was performed. The study population was stratified in the following subgroups: age (< 60 or ≥ 60 years old), sex (men or women), comorbidities (cardiovascular diseases, malignancies, gastrointestinal diseases, genitourinary disorders, endocrine/metabolic disorders, infectious and respiratory diseases), baseline eGFR (< 60 or ≥ 60 mL/min/1.73 m^2^).

For analysis and data calculation, we used R statistics (version 3.4.4, R Foundation for Statistical Computing Platform). A *p* value < 0.05 was considered as statistically significant.

### Ethical

The ethics committee of Fondazione Policlinico Universitario A. Gemelli IRCCS approved the research protocol (Prot. number 34327/18 ID 2210).

## Results

A total of 56,961 patients met our inclusion criteria. Table 1 presents the baseline characteristics of the study population. In total, 22,068 (38.7%) had cardiovascular diseases, 18,070 (31.7%) had malignancies, 9120 (16.0%) had gastrointestinal diseases, 6328 (11.1%) had genitourinary disorders, 10,070 (17.7%) had endocrine/metabolic disorders, 3049 (5.4%) infectious diseases, 7625 (13.4%) respiratory diseases, with a mean eGFR at hospital admission of 79.6 (SD 25.9) mL/min/1.73 m^2^ and a median Na value at hospital admission of 140 mEq/L (IQR 138.0–142.0, range lowest-highest 101.0–175.0 mEq/L) (Table 1).

**Table 1:** Baseline characteristics of the study population

	All *n* = 56,961	Normonatremia *n* = 44,178	Hyponatremia *n* = 8803	Hypernatremia *n* = 3980	*p* value
Age, years, mean (SD)	60.9 (18.0)	59.3 (18.0)	65.7 (17.4)	67.8 (15.6)	< 0.001
Males, *n* (%)	26,632 (46.8)	20,368 (46.1)	4411 (50.1)	1853 (46.6)	< 0.001
Charlson/Deyo score, *n* (%)					< 0.001
0	43,957 (77.2)	35,156 (79.6)	6138 (69.7)	2663 (66.9)	
1	9082 (15.9)	6477 (14.7)	1692 (19.2)	913 (22.9)	
2	2169 (3.8)	1456 (3.3)	470 (5.3)	243 (6.1)	
> 2	1753 (3.1)	1089 (2.5)	503 (5.7)	161 (4.0)	
Comorbidities, *n* (%)					
Cardiovascular	22,068 (38.7)	16,263 (36.8)	3795 (43.1)	2010 (50.5)	< 0.001
Malignancies	18,070 (31.7)	13,672 (30.9)	3166 (36.0)	1232 (31.0)	< 0.001
Gastrointestinal	9120 (16.0)	6757 (15.3)	1874 (21.3)	489 (12.3)	< 0.001
Genitourinary	6328 (11.1)	4593 (10.4)	1175 (13.3)	560 (14.1)	< 0.001
Endocrine/metabolic	10,070 (17.7)	7520 (17.0)	1709 (19.4)	841 (21.1)	< 0.001
Infectious	3049 (5.4)	1740 (3.9)	1008 (11.5)	301 (7.6)	< 0.001
Respiratory	7625 (13.4)	4871 (11.0)	1781 (20.2)	973 (24.4)	< 0.001
eGFR, mean (SD), mL/min/1.73 m^2^*	79.6 (25.9)	81.4 (25.2)	75.0 (28.1)	70.7 (25.5)	< 0.001
Na, median (IQR), mEq/L*	140.0 (138.0, 142.0)	140.0 (139.0, 142.0)	135.0 (132.0, 139.0)	144.0 (141.0, 146.0)	< 0.001
Na CV, median (IQR)	1.3 (0.7,1.9)	1.0 (0.5, 1.5)	2.3 (1.7, 3.0)	2.1 (1.5, 2.9)	< 0.001

*Value at hospital admission

In the study population, 44,178 stayed in a normonatremic condition while in 12,783 (22.4%) a dysnatremic status occurred (hyponatremia in 8803 [15.5%], hypernatremia in 3980 [7.0%]). Dysnatremic patients aged older (59.3 years [SD 18.0], 65.7 years [SD 17.4], 67.8 years [SD 15.6], in normo-, hypo-, hypernatremics, respectively, *p* < 0.001) with a higher prevalence in males (46.1%, 50.1%, 46.6%, in normo-, hypo- and hypernatremics, respectively, *p* < 0.001). Hyponatremia, and hypernatremia showed a worse comorbidity index, with a higher prevalence in cardiovascular diseases (36.8%, 43.1%, 50.5% in normo-, hypo- and hypernatremics, respectively, *p* < 0.001), malignancicies (30.9%, 36.0%, 31.0% in normo-, hypo- and hypernatremics, respectively, *p* < 0.001), genitourinary disorders (10.4%, 13.3%, 14.1% in normo-, hypo- and hypernatremics, respectively, *p* < 0.001), endocrine/metabolic disorders (17.0%, 19.4%, 21.1% in normo-, hypo- and hypernatremics, respectively, *p* < 0.001), infectious diseases (3.9%, 11.5%, 7.6% in normo-, hypo- and hypernatremics, respectively, *p* < 0.001), respiratory diseases (11.0%, 20.2%, 24.4% in normo-, hypo- and hypernatremics, respectively, *p* < 0.001). Lower eGFR was observed in patients with Na disorders (81.4 mL/min [SD 25.2], 75.0 mL/min [SD 28.1], 70.7 mL/min [SD 25.5] in normo-, hypo- and hypernatremics, respectively, *p* < 0.001).

Coefficient of Na variation (Na CV) was used to describe Na fluctuation in the study cohort. Patients with higher Na variability showed worse comorbidty index and higher prevalence in comorbidities. Older age and lower baseline eGFR was also observed in subjects with higher Na fluctuations (Table 2).

**Table 2:** Baseline characteristics of the study population stratified by quartile of Na coefficient of variation (CV)

	1st CV ≤ 0.71 *n* = 14,580	2nd 0.71 < CV ≤ 1.25 *n* = 14,037	3rd 1.25 < CV ≤ 1.94 *n* = 14,206	4th CV > 1.94 *n* = 14,138	*p* value
Age, years, mean (SD)	57.8 (18.3)	60.5 (17.9)	62.1 (17.3)	63.2 (18.0)	< 0.001
Men, *n* (%)	6587 (45.2)	6806 (48.5)	6925 (48.7)	6314 (44.7)	< 0.001
Charlson/Deyo score, *n* (%)					< 0.001
0	11,771 (80.7)	10,869 (77.4)	10,754 (75.7)	10,563 (74.7)	
1	2063 (14.1)	2232 (15.9)	2375 (16.7)	2412 (17.1)	
2	411 (2.8)	531 (3.8)	585 (4.1)	642 (4.5)	
> 2	335 (2.3)	405 (2.9)	492 (3.5)	521 (3.7)	
Comorbidities, *n* (%)					
Cardiovascular	5130 (35.2)	5424 (38.6)	5837 (41.1)	5677 (40.2)	< 0.001
Malignancies	4151 (28.5)	4440 (31.6)	4808 (33.8)	4671 (33.0)	< 0.001
Gastrointestinal	2307 (15.8)	2229 (15.9)	2342 (16.5)	2242 (15.9)	0.363
Genito/urinary	1408 (9.7)	1502 (10.7)	1617 (11.4)	1801 (12.7)	< 0.001
Endocrine/metabolic	2466 (16.9)	2441 (17.4)	2655 (18.7)	2508 (17.7)	0.001
Infectious	496 (3.4)	632 (4.5)	820 (5.8)	1101 (7.8)	< 0.001
Respiratory	1277 (8.8)	1654 (11.8)	2122 (14.9)	2572 (18.2)	< 0.001
eGFR, mean (SD), mL/min/1.73 m^2^*	83.0 (25.0)	80.1 (25.5)	78.1 (25.8)	77.3 (27.1)	< 0.001
Na, median (IQR), mEq/L*	140.0 (139.0, 142.0)	140.0 (138.0, 142.0)	140.0 (138.0, 142.0)	139.0 (136.0, 142.0)	< 0.001
Na CV, median (IQR)	0.5 (0.0, 0.5)	1.0 (1.0, 1.1)	1.5 (1.5, 1.7)	2.5 (2.1, 3.1)	< 0.001

*Value at hospital admission

### Sodium and AKI

#### Dysnatremia and AKI

During 1541 years of follow-up, the outcome occurred in 1450 (2.5%, incidence rate 940.9 per 1000 person-year).

A dysnatremic condition was associated with increased risk of AKI development (Table 3). Patients with dysnatremia or patients who developed dysnatremia had a higher risk of AKI occurrence with an HR, in multivariable adjusted model, of 1.87 (95% CI 1.61, 2.16, *p* < 0.001), HR 1.67 (95% CI 1.41, 1.98, *p* < 0.001), in hyponatremia and hypernatremia, respectively.

**Table 3:** Association of dysnatremia with AKI development

	Overall	Normonatremia	Hyponatremia	Hypernatremia
No. of patients	56,961	44,178	8803	3980
Person-years	1541	1167	269	106
Time to AKI, median (IQR), days	–	–	8.0 (10.0)	7.0 (8.0)
Kidney outcome				
AKI (*N*, %)	1450 (2.5)	864 (2.0)	381 (4.3)	205 (5.2)
AKI per 1000 person-year	940.9	740.4	1416.4	1934.0
Cause-specific hazard model				
HR (95% CI	–	1.00 (Reference)	1.98 (1.76, 2.24) *p* < 0.001	2.69 (2.31, 3.13) *p* < 0.001
HR (95% CI)*	–	1.00 (Reference)	1.87 (1.61, 2.16) *p* < 0.001	1.67 (1.41, 1.98) *p* < 0.001

*Adjusted for: age, sex, comorbidities, Na value at hospital admission, eGFR at baseline

#### Sodium variability and AKI

In Table 4, we investigated the association between Na variability and AKI. Unadjusted regression models suggested a higher risk for AKI development in the 2nd (OR 1.45, 95% CI 1.21, 1.73, *p* < 0.001), 3rd (OR 2.08, 95% CI 1.77, 2.47, *p* < 0.001) and 4th (OR 2.70, 95% CI 2.30, 3.18, *p* < 0.001) quartile of Na CV. This association was also observed in the multivariable adjusted model (Model 2) with an OR of 1.25 (95% CI 1.04, 1.50, *p* = 0.017), an OR of 1.66 (95% CI 1.40, 1.97, *p* < 0.001) and an OR of 2.12 (95% CI 1.79, 2.51, *p* < 0.001) in the 2nd, 3rd and in the 4th quartile of Na CV respectively with a significant linear trend across all quartiles (*p* for trend < 0.001). Of note, even after adjustment for Na lowest and highest peak value (Model 3) the significant and independent relationship was confirmed.

**Table 4:** Association between Na variability (CV) and AKI development

	No. of events (%)	Model 1		Model 2		Model 3	
		OR (95% CI)	*p* value for trend	OR (95% CI)	*p* value for trend	OR (95% CI)	*p* value for trend
Q1 CV ≤ 0.71	209 (1.4)	1.00 (Reference)	< 0.001	1.00 (Reference)	< 0.001	1.00 (Reference)	< 0.001
Q2 0.71 < CV ≤ 1.25	289 (2.1)	1.45 (121, 1.73) *p* < 0.001		1.25 (1.04, 1.50) *p* = 0.017		1.16 (0.97, 1.40) *p* = 0.115	
Q3 1.25 < CV ≤ 1.94	418 (2.9)	2.08 (1.77, 2.47) *p* < 0.001		1.66 (1.40, 1.97) *p* < 0.001		1.41 (1.18, 1.70) *p* < 0.001	
Q4 CV > 1.94	534 (3.8)	2.70 (2.30, 3.18) *p* < 0.001		2.12 (1.79, 2.51) *p* < 0.001		1.53 (1.24, 1.91) *p* < 0.001	

Model 1: Unadjusted model

Model 2: Multivariable adjusted logistic regression. Adjusted for age, sex, comorbidities, Na value at hospital admission, eGFR baseline

Model 3: Multivariable adjusted logistic regression. Adjusted for age, sex, comorbidities, Na value at hospital admission, eGFR baseline, Na lowest and highest peak value

#### Subgroup analysis

We evaluated the modification effects of subgroups on the relationship between dysnatremia and AKI development performing subgroup analysis (Table 5). A significant interaction was observed in dysnatremic patients older than 60 years, with cardiovascular and endocrine/metabolic disorders; there was also an effect modification only in hyponatremic patients male, with gastrointestinal diseases, genito/urinary disorders and eGFR < 60 mL/min.

**Table 5:** Subgroup associations of dysnatremia with AKI development

	AKI (hyponatremia sample)		AKI (hypernatremia sample)	
	HR (95% CI)	*p* for interaction	HR (95% CI)	*p* for interaction
Age ≥ 60	4.32 (3.26, 5.71)	< 0.001	4.26 (2.80, 6.48)	0.003
Age < 60	1.53 (1.34, 1.76)		2.14 (1.81, 2.52)	
Male	2.58 (2.12, 3.14)	< 0.001	3.23 (2.53, 4.13)	0.075
Female	1.65 (1.41, 1.92)		2.43 (2.00, 2.95)	
Cardiovascular	3.35 (2.73, 4.10)	< 0.001	4.39 (3.36, 5.73)	< 0.001
No cardiovascular	1.42 (1.22, 1.66)		1.85 (1.53, 2.23)	
Malignancies	1.85 (1.60, 2.15)	0.069	2.73 (2.30, 3.26)	0.652
No malignancies	2.36 (1.91, 2.92)		2.52 (1.84, 3.45)	
Gastrointestinal	1.85 (1.61, 2.11)	0.005	2.68 (2.28, 3.15)	0.960
No gastrointestinal	2.93 (2.19, 3.91)		2.71 (1.97, 4.40)	
Genito/urinary	2.04 (1.78, 2.34)	0.031	2.75 (2.32, 3.27)	0.089
No genito/urinary	1.47 (1.13, 1.92)		2.00 (1.44, 2.77)	
Endocrine/metabolic	2.25 (1.96, 2.59)	< 0.001	3.10 (2.61, 3.70)	0.001
No endocrine/metabolic	1.34 (1.05, 1.71)		1.71 (1.25, 2.34)	
Infectious	2.08 (1.83, 2.37)	0.085	2.59 (2.20, 3.05)	0.314
No infectious	1.46 (1.00, 2.14)		3.29 (2.12, 5.11)	
Respiratory	2.21 (1.93, 2.54)	< 0.001	2.71 (2.24, 3.28)	0.100
No respiratory	1.23 (0.96, 1.59)		2.06 (1.58, 2.69)	
eGFR ≥ 60	1.37 (1.17, 1.61)	< 0.001	1.95 (1.61, 2.36)	0.123
eGFR < 60	2.28 (1.89, 2.73)		2.51 (1.94, 3.24)	

#### Sensitivity analysis

As sensitivity analysis a subdistribution hazard model between AKI development and dysnatremic groups was built (Supplemental Table 1) using death as competitive risk. The relationship between the outcome of interest and the exposures was confirmed, with a significant and independent increase risk of AKI development in dysnatremic groups (HR 1.84, 95% CI 1.58, 2.16, *p* < 0.001, HR 1.62, 95% CI 1.35, 1.95, *p* < 0.001, respectively in hyponatremia and hypernatremia condition).

## Discussion

Our study demonstrates a strong and independent association between Na disturbances and Na variability with the risk of AKI development. Dysnatremia, hyponatremia or hypernatremia are significantly associated with acute kidney injury and higher Na variability (CV) predicts AKI development during hospital stay.

AKI is a complex clinical syndrome characterized by a sudden reduction in renal function and defined as an increase (absolute or relative) of creatinine levels or a reduction in urinary output [24]. The high burden on in-hospital patient prognosis [25, 26] justifies the medical and scientific interest in this clinical syndrome. Since the kidney is the main organ engaged in fluid and electrolyte homeostasis, it is not surprising that renal dysfunctions are frequently associated with water imbalance and so alterations in serum electrolyte levels. In particular, hypo and hypernatremia conditions, often observed in the hospitalized population [1], have been related to kidney injury [11–15].

A growing body of recent evidences highlight the relationship between disorders of metabolism and electrolyte homeostasis and strong outcomes [8, 27, 28]. Several studies have described the association between Na balance and kidney function [11–13] however, a few of them have considered dysnatremia as predictor of AKI [14, 19] and none has investigated Na variability as a marker in kidney injury development.

During 1541 person-years of follow-up, AKI was observed in 1450 (2.5%) patients (incidence rate 940.9 per 1000 person-year); 588 (40.4%) of them showed a dysnatremia condition (at hospital admission or during hospital stay) before AKI development. As clearly demonstrated by our results, Na disturbances were strongly and independently associated with in-hospital AKI. Of note, higher Na fluctuations were linearly related to an increased risk of AKI independently of Na peak value.

Many pathological conditions may share dysnatremia and AKI such as volume overload or depletion, heart and liver diseases, infectious diseases and partly explain our findings. Under specific conditions, such as cardiovascular diseases, cardio-renal syndrome, gastrointestinal diseases or malignancies, hyponatremia has been associated with AKI development [11–13]. According to our findings, older patients, with cardiovascular diseases and endocrine/metabolic disorders with dysnatremia were especially associated with increased risk of AKI.

However, an independent relationship between Na disturbances and Na variability emerges from our study. Recently Lee et al., focusing only on an hyponatremic hospitalized population, described an independent correlation between dysnatremia and AKI development, where pre-existing hyponatremia increased the risk of AKI by 30% [19].

Using our retrospective cohort, we decided to go further these findings. Not only pre-existing hyponatremia condition was associated with kidney injury, but also hypernatremia demonstrated as a potential independent predictor of AKI. Interestingly, higher Na variability was often observed before kidney damage. From this point of view, since serum creatinine is such a poor sensitive marker of kidney damage, higher Na fluctuations may anticipate worsening of renal function.

An appropriate question should be whether dysnatremia is a simple bystander or contributing factor of AKI. Dysnatremia and kidney injury may be two different manifestations of a common underlying disease or reflect the severity of the illness and comorbidities of the patient. Volume depletion that generally accompanies hypernatremia is frequently recognized as a common cause of AKI development. On the other hand, Na dilution (or true Na depletion due to diuretics) can be observed in systemic disorders (i.e., heart, liver failure) causing AKI, characterized by increased extracellular volume.

However, according to our results, although we could not exclude residual confounding due to the retrospective nature of the study, a direct pathogenic relationship between Na imbalance and kidney injury might be hypothesized. Concerning this, it is interesting to note the median time between dysnatremia and the onset of AKI (7–8 days), as reported on Table 3. An interesting finding in our study, Na variability and kidney injury were associated independently from Na peak value (highest or lowest), in accordance with what has been shown by other Authors [8, 27], suggesting a potentially more damaging role of Na rapid variations rather than Na absolute value. Evidence suggests that osmotic stress can cause cellular damage, even if we do not know the exact mechanism at kidney level. The direct effect of serum sodium concentration and variation on extracellular tonicity is well known. Serum sodium fluctuations induce water shift in and out of the cells threatening their survival [29–31]. A wide variety of experimental models have demonstrated that osmo-stress can evoke multiple apoptotic pathways, inhibit anti-apoptotic gene expression, induce multiple cytokines and reactive oxygen species generation [32–34]. Even if we do not know the exact mechanism of osmotic damage at kidney level, it would be consistent with the reported evidence; however, it has yet to be elucidated in detail. Further studies, with a prospective design, are required to carefully explore and confirm such causal association.

Several study limitations have to be reported such as a retrospective and monocentric design, the use of ICD-9-CM codes to identify comorbid conditions, unavailability of information on chronic or acute therapy administered during hospital stay. However, the use of Charlson/Deyo comorbidity index score to account for patient comorbidity (a validated index of diseases severity based on ICD-9-CM codes), a wide general in-hospital cohort and a creatinine-based definition of in-hospital AKI give strength to our results.

To our knowledge, this is the first study that has widely analyzed the association between Na disorders and kidney injury demonstrating that: (i) dysnatremia is a common condition that involves AKI patients; and (ii) high Na variability might be considered a good biological marker that anticipates kidney injury development.

## Electronic supplementary material

Below is the link to the electronic supplementary material.

Supplementary file1 (DOCX 15 kb)
