# Supplementary material for: Serum sodium variability and acute kidney injury: a retrospective observational cohort study on a hospitalized population
Source: Intern Emerg Med. 2020 Aug 9;16(3):617–24. doi: 10.1007/s11739-020-02462-5 (PMC8049924; doi:10.1007/s11739-020-02462-5)
Supplement: Supplementary file 1 — Supplementary file1 (DOCX 15 kb) [file 11739_2020_2462_MOESM1_ESM.docx]

**Supplemental Table 1. Association of dysnatremia with AKI development.**

Subdistribution hazard model

|  | **Normonatremia** | **Hyponatremia** | **Hypernatremia** |
| --- | --- | --- | --- |
| No. of patients  **In-hospital outcome**  Death  AKI  **Subdistribution hazard model**  HR (95% CI)  HR (95% CI)* | 44,178  195 (0.4)  864 (2.0)  1.00 (Reference)  1.00 (Reference) | 8,803  304 (3.5)  381 (4.3)  1.95 (1.73, 2.21)  *p*<0.001  1.84 (1.58, 2.16)  *p*<0.001 | 3,980  194 (4.9)  205 (5.2)  2.62 (2.25, 3.05)  *p*<0.001  1.62 (1.35, 1.95)  *p*<0.001 |

*Adjusted for: Age, Sex, Comorbidities, Na value at hospital admission, eGFR baseline
